# Dr. Clifford Finley Stipp

**Published:** 1901-12-15

**Authors:** 


### DR. CLIFFORD FINLEY STIPP.

Dr. Clifford Finley Stipp, the only son of J. A. Stipp, of Toledo, Ohio, died November 21, 1901, of typhoid pneumonia, after a severe illness. He graduated from the College of Dental Surgery of the University of Michigan in June, 1899, and has since been engaged in practice at Toledo. He was associated in business with his father, to whom his death is a severe loss. He was a well educated and highly accomplished young man, and would no doubt have made a good citizen and a faithful practitioner, and his untimely taking off is to be greatly deplored. The father has the sympathy of a large circle of professional and social acquaintances and friends.

